# Effects of the cage height and positioning on clinical and radiographic outcome of lateral lumbar interbody fusion: a retrospective study

**DOI:** 10.1186/s12891-022-05893-7

**Published:** 2022-12-09

**Authors:** Changyuan Wu, Hanming Bian, Jie Liu, Dong Zhao, Haiyun Yang, Chao Chen, Xun Sun, Binggang Guan, Guiming Sun, Gang Liu, Baoshan Xu, Xinlong Ma, Zheng Wang, Qiang Yang

**Affiliations:** 1 Departments of Orthopaedics, Tianjin Hospital, Tianjin Medical University, Tianjin, China; 2 Department of Spine Surgery, Tianjin Hospital, Tianjin University, No. 406 Jiefangnan Road, Hexi District, Tianjin, 300211 China; 3 Department of Orthopedics, No.1 Medical Center of Chinese PLA General Hospital, Beijing, China

**Keywords:** Lateral lumbar interbody fusion, Cage position, Cage height, Cage subsidence

## Abstract

**Background:**

The proper cage positioning and height in lateral lumbar interbody fusion (LLIF). This study evaluated their effects on clinical and radiographic outcome measures in patients undergoing LLIF.

**Methods:**

This single-center retrospective study analyzed the characteristics and perioperative data of patients who underwent LLIF between January 2019 and December 2020. Radiographic (lumbar lordosis [LL], foraminal height, disc height [DH], segmental angle [SA], cross-sectional area [CSA] of thecal sac) and clinical (Oswestry Disability Index and Visual Analog Scale) outcomes were assessed preoperatively, postoperatively, and at the last follow-up. The effects of cage height and positioning on these parameters were also investigated.

**Results:**

With a mean follow-up of 12.8 months, 47 patients with 70 operated level were analyzed. Data demonstrated that postsurgical clinical and radiographic outcome measures were significantly better than before surgery(*P* < 0.05). Cage height and positioning showed no significant difference with regarding to clinical outcome(*P* > 0.05). Subgroup analysis of the cage positioning showed that DH and SA were better restored by the final follow-up in patients with anteriorly placed cages than those with posteriorly placed cages (*P* < 0.05). Cages of posterior position showed significantly upgrading cage subsidence (*P* = 0.047). Cage height subgroup analysis showed that the preoperative forminal height, DH, and SA in the 11-mm cage group were significantly lower than in the 13-mm cage group; however, these parameters were comparable in the two groups postoperatively and at the final follow-up (*P* > 0.05). Furthermore, the postoperative and final follow-up degrees of DH, SA, and LL have improved in the 11-mm cage group more than the 13-mm cage group. The preoperative, postoperative, and final follow-up LL values in the 11-mm cage group were lower than in the 13-mm cage group(*P* < 0.01).

**Conclusions:**

Cage height and positioning did not affect the clinical outcomes in the present study. Cages in anterior position showed better restoration in DH, SA and decreased the incidence of cage subsidence. A comparable radiographic outcome can be achieved by inserting an appropriate cage height based on preoperative radiography.

## Background

Nearly 20 years since it was first introduced [1], lateral lumbar interbody fusion (LLIF) is still gaining popularity. It represents an alternative minimally invasive approach that allows access to the intervertebral disc space and vertebral bodies via a retroperitoneal transpsoas approach [1, 2]. This procedure gains pervasive attention because it allows lateral access to the lumbar disc through a small incision in the flank and insertion of a large interbody graft that indirectly decompresses neurological tissue by expansion of disc and foraminal height [3–6]. Moreover, it obviates the need for mobilization of the most abdominal content and does not require the assistance of a general surgeon [6, 7]. Additionally, this approach preserves the inherent biomechanical integrity of the motion segment through maintenance of the longitudinal ligaments of the spine and posterior musculature [8]. The lordotic cages utilized in LLIF effectively restore spinopelvic parameters and disc angles [9]. The major limitations of the LLIF include neurovascular complications, anatomical limitations, cage subsidence and loss of correction, declining the potential to restore spinal biomechanics sustainably [6, 10–12].

Previous studies have shown that the cage height and position implanted in transforaminal lumbar interbody fusion (TLIF) are important [13, 14]. However, there is no consensus in the cage proper positioning and height in LLIF. According to Alimi et al. [15], the cage position and height result in no significant difference in radiographic parameters, results corroborated by Limthongkul et al. [16] and Kepler et al. [4]. However, Ebata et al. [17] reported the central placement of cage may be advantageous for indirect decompression. In addition, a study reported by Park et al. [18] demonstrated that the cage position in extreme lateral interbody fusion (XLIF) could lead to the increase of segmental angle and anterior disk height and found that the anterior one-third of disk space was beneficial for better restoration of the segmental angle if the cage height was large enough. Since the proper height and position of the LLIF cages were still in debate, the main goal of this study was to evaluate the impact of cage position and height on clinical and radiological outcomes of LLIF procedure in the treatment of degenerative lumbar pathologies.

## Material and methods

Consecutive patients treated with LLIF between January 2019 and December 2020 were analyzed retrospectively. Information on patient characteristics, preoperative data, the surgical procedure performed, and clinical and radiographic outcomes, and average duration of follow-up were extracted from the medical records. Patients were eligible for study inclusion if they were ≥ 18 years old, diagnosed with lumbar degenerative diseases, unresponsive to conservative treatment for at least six months, and followed up for at least six months postoperatively. Indications for LLIF included low-grade lumbar spondylolisthesis (Meyerding grade I/II), lumbar spinal stenosis, degenerative scoliosis, and degenerative disc disease. Patients were excluded if they had undergone previous lumbar fusion surgery, lumbar spondylolisthesis of grade III and above, trauma, or tumors. The study met the guidelines of the responsible local governing agency and complied with the principles of the Declaration of Helsinki. The patients and their families were informed that the data from the cases would be submitted for publication, and written consent was obtained.

The techniques used in this study followed those described by Zhengkuan et al. [19] and the details can be found in their publication. And the intraoperative electromyography monitoring was not utilized due to the operative approach under direct visualization and direct injury to the subcostal, iliohypogastric, and genitofemoral nerves could be avoided. Standard left lateral transpsoas approach was performed, other than patients with degenerative scoliosis, concave side was preferred. All patients were fitted with an 8-degree lordotic intervertebral poly-ether-ether-ketone (PEEK) cage (DePuy Synthes GmbH, Oberdorf, Switzerland). The cages were 50 or 55 mm in length, 22 mm in width and 11 or 13 mm in height.

**Fig. 1:**
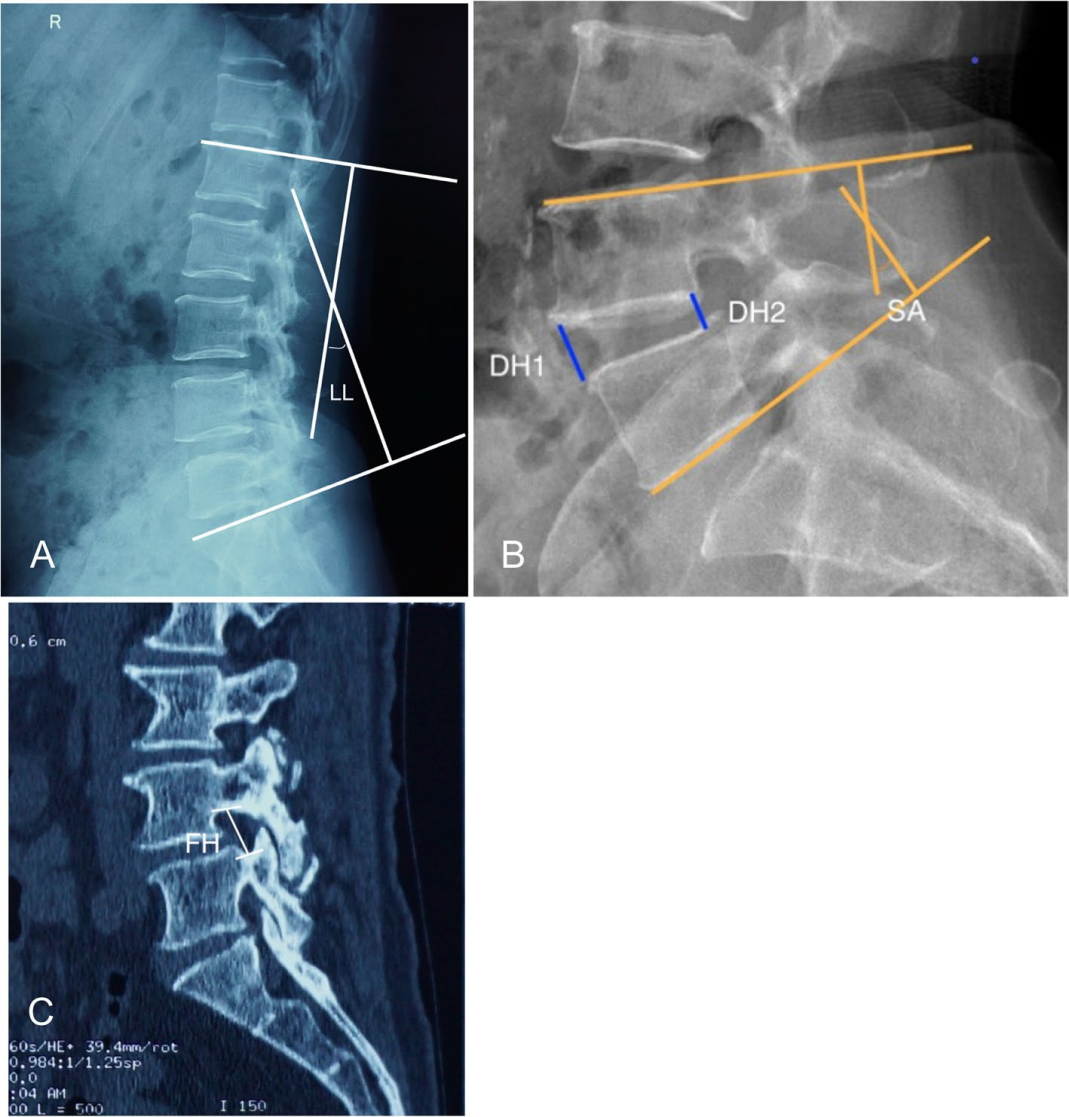
Radiological measurements of LL(**A**), DH(**B**), and SA(**B**) on lateral X-ray. FH was measured on parasagittal reconstruction CT(**C**). LL, lumbar lordosis; DH, disc height; SA, segmental angle; FH, foraminal height

**Fig. 2:**
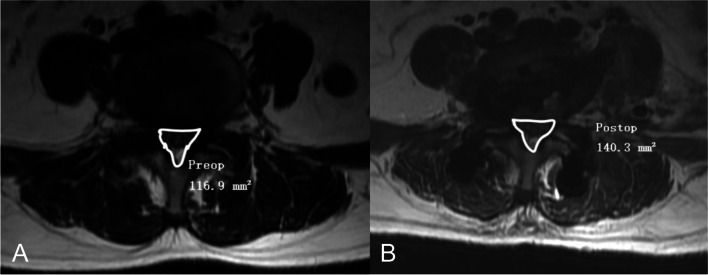
An example of measurement of CSA of the thecal sac preoperatively (**A**) and postoperatively (**B**). CSA, cross-sectional area

Clinical results of the patients were assessed using the Oswestry disability index (ODI) and visual analog scale (VAS) for back pain and leg pain. Radiological parameters were measured on the preoperative, immediate postoperative and final follow-up images. CT scans, plain radiograph, and magnetic resonance (MR) images (first, second, and third priority, respectively) were used for radiological measurements, depending on each patient’s available imaging studies. Lumbar lordosis (LL) was measured between the cephalad endplate of L1 and the cephalad endplate of the sacrum [15] (Fig. 1A). Disc height (DH) was measured on lateral views by determining the mean value of the anterior and posterior disc heights for each level [15, 16] (Fig. 1B). Segmental angle (SA) was defined as the Cobb’s angle between the upper endplate and lower endplate at the operated level [15] (Fig. 1B). Foraminal height (FH) was defined as the mean value of the right and left foraminal height on the CT scans [16] (Fig. 1C). Cross-sectional area (CSA) of thecal sac was measured at the facet joint level on axial cut T2- weighted MRI [16] (Fig. 2). Fusion was defined as bridging bone connecting adjacent vertebral bodies either through the implant or around it based on radiographic evaluation. The fusion pattern was categorized based on CT scans reported by Proietti et al. [20]: anterior only, posterior only, non-fusion, and circumferential fusion.

Continuous variables are presented as means ± standard deviations (SDs) after confirming normal distribution using the Shapiro-Wilk test. Differences were assessed using the independent-samples or paired-samples *t*-test, as appropriate. Correlations between continuous variables were examined using Pearson’s correlation coefficient. Percentages were calculated for the categorical variables. Analysis was considered statistically significant with *P*-values<0.05. All analyses were performed using SPSS version 24.0 (IBM Corp, Armonk, NY, USA).

## Results

In total, 47 consecutive patients with 70 spinal motion segments were included in this study. Patients’ characteristics are summarized in Table 1. The most common indications for LLIF were spinal stenosis (47%), spondylolisthesis (38%), degenerative scoliosis (9%), and degenerative disc disease (6%).

**Table 1:** Patients’ demographic data

Variables	*n* = 47
Gender(M:F)	7:40
Age (years)	58.8 ± 7.4
BMI	25.1 ± 3.2
Follow-up (months)	12.8 ± 6.3
Indications	
Lumbar spinal stenosis	22
Spondylolisthesis	18
Degenerative disc disease	3
Degenerative scoliosis	4
Comorbidity	
Hypertension	9
Heart disease	4
Cerebral vascular disease	1
Diabetes Mellitus	5

### Surgical outcomes

**Fig. 3:**
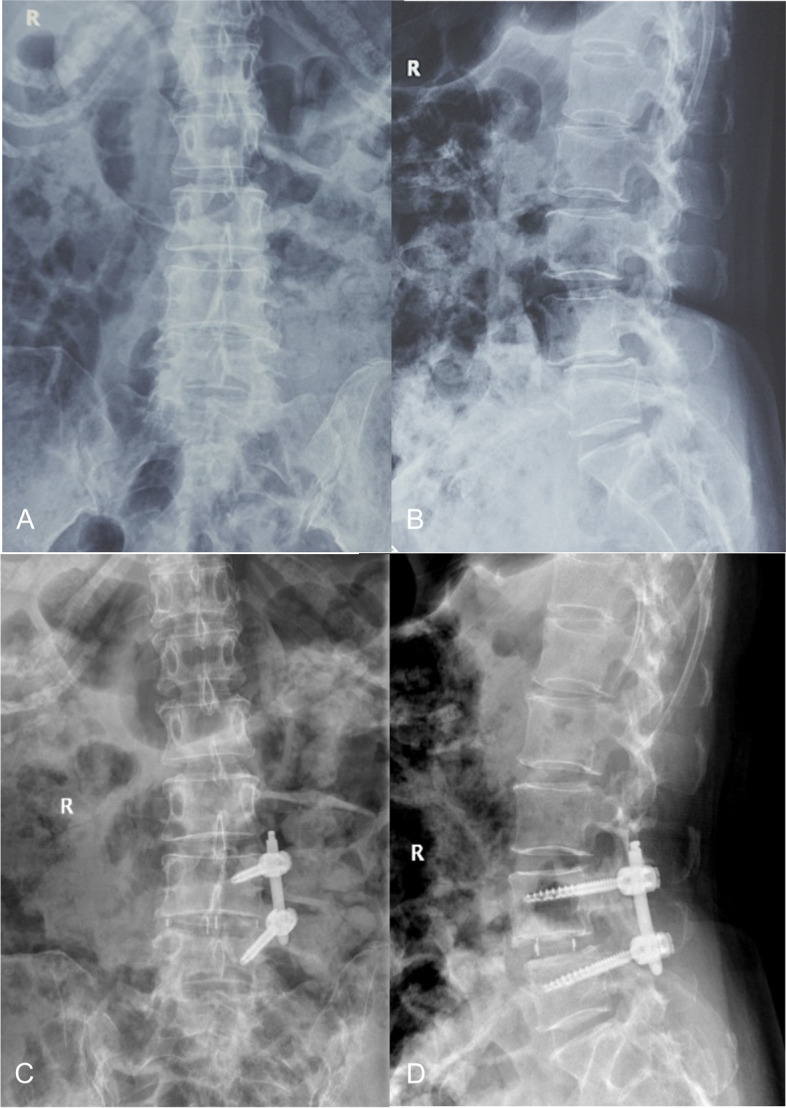
A 57-year-old female patient at the time of presentation with lumbar spondylolisthesis. Image workup demonstrated responsible lesion at L4/5. The L4/5 were treated with unilateral pedicle screws, and the cage was implanted anteriorly. At the final follow-up, her ODI, VAS lumbar and leg pain scores had decreased from 50%, 5, 5 to 30%, 2, 3 respectively. Preoperative anteroposterior (**A**) and lateral (**B**) radiographs show typical radiological findings of lumbar spondylolisthesis. Anteroposterior (**C**) and lateral (**D**) radiographs obtained immediate postoperatively and the latter illustrated an anterior cage position

**Fig. 4:**
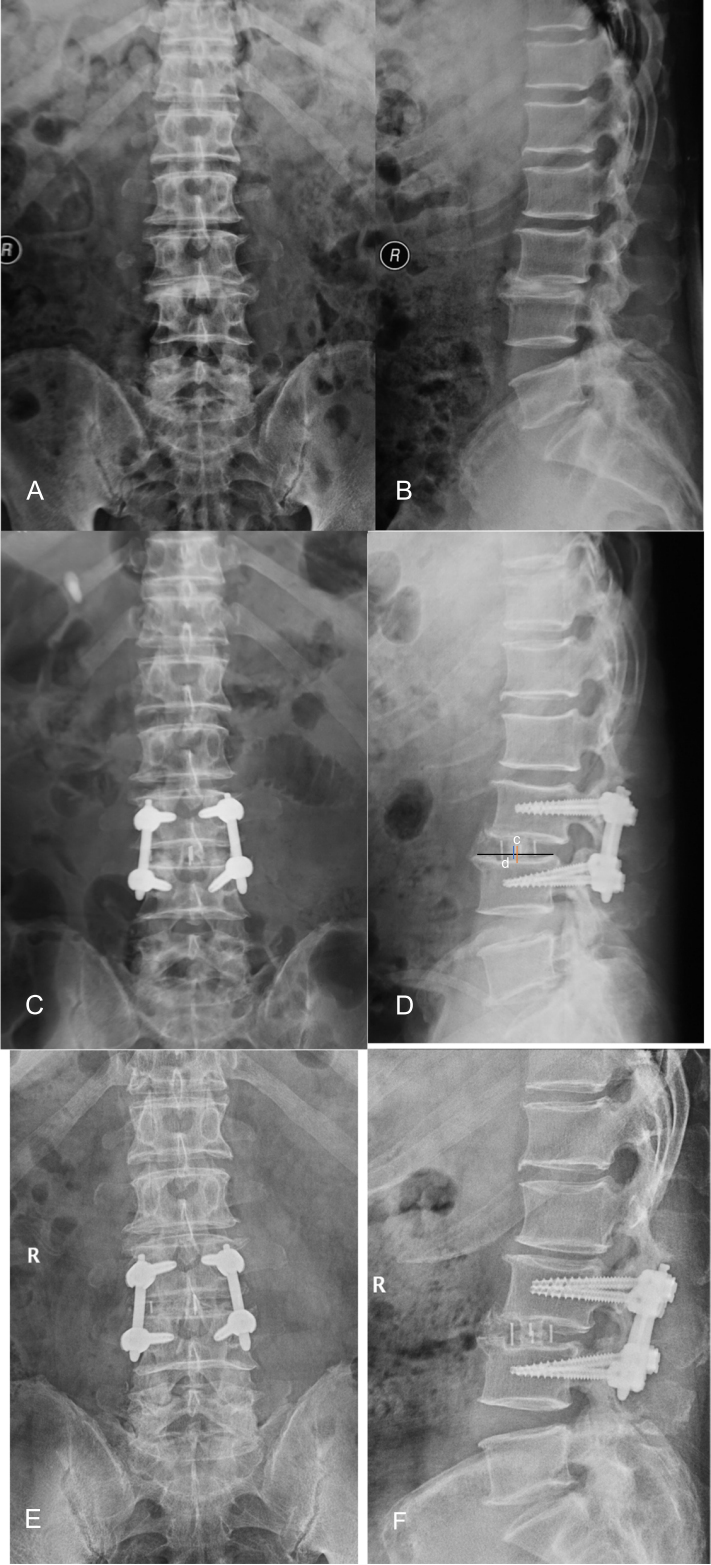
A 63-year-old male patient at the time of presentation with degenerative disc disease. Image workup demonstrated responsible lesion at L3/4. He was treated with XLIF. The L3/4 segments were treated with instrumentation using pedicle screws, and the position of cage was posterior. At the final follow-up, his ODI, VAS lumbar and leg pain scores had decreased from 20%, 5, 3 to 19%,3, 2 respectively. Cage subsidence was found in this case. Preoperative anteroposterior (**A**) and lateral (**B**) radiographs show typical radiologic findings of degenerative disc disease. Anteroposterior (**C**) and lateral (**D**) radiographs obtained immediate postoperatively and the latter illustrated a posterior cage position(c) posterior from the midpoint of inferior endplate(d). Anteroposterior (**E**) and lateral (**F**) radiographs acquired at final follow-up showed typical cage subsidence

The most common surgical level was L4–5(57%), followed by L3–4(24%) and L2–3(13%; Table 2). Thirty-two patients underwent single-level surgery, while 17 patients underwent multilevel LLIF procedures (2-level, 9 cases; 3-level, 4 cases; 4-level, 4 cases; Table 2). As is illustrated in Table 2, we approached from the left side and used supplemental instrumentation in all cases; The instrumentation type was selected at the surgeon’s discretion. In general, pedicle screws and rods were used in cases where posterior elements were thought to not have sufficient stability, and in mild deformity cases. Among them, 24.3% were instrumentation on unilateral (Fig. 3), and 75.7% were bilateral instrumentation (Fig. 4). Cages of 11-mm (49% of the levels) and 13-mm (51% of the levels) in height were used. Cages were implanted either in an anterior position(69% of the levels), which was defined by radiographic position of the cage midpoint anteriorly toward the midpoint of the inferior endplate or in a posterior position(31% of the levels) (Fig. 4), which was defined by radiographic position of the cage midpoint anteriorly toward the midpoint of the inferior endplate [15, 16] (Fig. 3). The mean operation time was 107.2 ± 22.8 min, and the average estimated blood loss was 143.0 ± 71.9 mL.

**Table 2:** Characteristics of LLIF Procedure of 70 Functional Spinal Segments

Parameters	XLIF	*N* = 70
Spinal Levels	L1–2	4
	L2–3	9
	L3–4	17
	L4–5	40
No. Levels	1	32
	2	18
	3	12
	4	8
Instrumentation	Unilateral	17
	Bilateral	53
Average OR time (min)		107.2 ± 22.8
Average EBL (ml)		143.0 ± 71.9
Cage Height (mm)	11	34
	13	36
Cage Position	Anterior	48
	Posterior	22
Cage Right-Left Width (mm)	50	46
	55	24
Postoperative Hospital Stays (days)		5.1 ± 3.2

### Radiographic analysis

All patients underwent plain radiographic examination at the last follow-up; however, 21 patients with 34 operated levels did not undergo MR and CT scanning for personal reasons. As the FH and CSA were obtained from the CT and MR images, these were available at the last follow-up for 26 cases with 36 operated levels. The mean follow-up duration for radiological outcomes was 12.8(range 6–28) months. As shown in Table 3, LL, DH, FH, SA, CSA values immediately after surgery and at the last follow-up were significantly better than preoperatively. The fusion rate at the last follow-up was 84.3% (59/70), as evaluated by plain radiography. Based on CT scans obtained at the last follow-up, 14 spinal segments (38.9%) were fused anteriorly only, 22 (61.1%) underwent circumferential fusion, and none underwent only posterior fusion or non-fusion.

**Table 3:** Overall radiographic and clinical outcome of LLIF

Parameters	Preoperative	Postoperative	Postoperative-Preoperative (P)	Final Follow-Up	Final Follow-Up-Preoperative (P)
Lumbar Lordosis(°)	36.3 ± 11.5	38.5 ± 6.8	<0.01^*^	38.4 ± 6.4	<0.01^*^
Disc Height (mm)	6.9 ± 2.4	8.8 ± 1.4	<0.01^*^	8.8 ± 1.6	<0.01^*^
Foraminal Height (mm)	14.2 ± 1.6	15.8 ± 2.8	<0.01^*^	16.6 ± 1.0^a^	<0.01^*^
Segmental Angle(°)	5.6 ± 3.0	11.2 ± 5.0	<0.01^*^	6.9 ± 1.8	<0.01^*^
Cross-Sectional Area of Thecal Sac (mm^2^)	136.7 ± 23.0	178.2 ± 15.6	<0.01^*^	173.5 ± 9.0^a^	<0.01^*^
VAS Back Pain	4.8 ± 1.1	2.4 ± 1.0	<0.01^*^	2.46 ± 0.8	<0.01^*^
VAS Leg Pain	4.5 ± 1.0	1.9 ± 0.9	<0.01^*^	1.8 ± 0.8	<0.01^*^
ODI	44.3% ± 12.0%	19.1% ± 8.6%	<0.01^*^	23.8% ± 7.2%	<0.01^*^

ODI indicates Oswestry Disability Index, *VAS* Visual analog scores

* *P*<0.05 is considered statistically significant

^a^Corresponding radiographic parameters obtained from 26 cases with 36 operated levels with CT and MR images at the final follow-up

Comparison between the cage positions found significant differences in preoperative DH and FH, and postoperative SA, DH, and disc height subsidence, and fusion patterns (Tables 4 and 5). The improvement from baseline to 12-month postoperative DH and SA with a cage in anterior position was significantly greater than with cage in the posterior position (2.2 mm vs. 1.2 mm, *P* = 0.043, 1.8° vs. 0.4°, *P* = 0.025, respectively). Furthermore, cage subsidence in the posterior position was significantly greater than in anterior position (−0.35 mm vs. 1.3 mm, *p* = 0.043; Table 4 and Fig. 4).

**Table 4:** Radiographic and clinical outcome of cage position, cage height subgroup analysis

Parameters	Cage position			Cage height		
	Anterior(*N* = 48)	Posterior(*N* = 22)	Significance(P)	11 mm(*N* = 34)	13 mm(*N* = 36)	Significance(P)
**Regional Sagittal Lumbar Lordosis (°)**						
Preoperative Value	35.1 ± 12.6	39.1 ± 8.2	0.179	32.2 ± 14.2	40.2 ± 14.2	0.004*
Postoperative Value	27.7 ± 15.3	32.1 ± 14.0	0.288	26.8 ± 14.7	31.3 ± 15.1	0.006*
Follow-Up Value	37.9 ± 7.4	39.8 ± 5.2	0.315	36.2 ± 7.8	40.6 ± 4.8	0.021*
Preoperative To Postoperative Increase	2.8 ± 6.4	0.7 ± 3.5	0.149	4.0 ± 7.2	0.5 ± 2.9	0.011*
Preoperative To Latest of Follow-Up Increase	2.8 ± 6.5	0.4 ± 4.0	0.128	4.3 ± 7.3	−0.1 ± 2.9	0.002*
**Foraminal Height (mm)**						
Preoperative Value	13.9 ± 1.5	14.8 ± 1.5	0.029*	13.7 ± 1.7	14.6 ± 1.3	0.022*
Postoperative Value	15.7 ± 2.8	16.1 ± 2.8	0.514	15.5 ± 3.0	16.1 ± 2.6	0.375
Follow-Up Value^a^	16.5 ± 1.2	16.7 ± 0.8	0.707	16.5 ± 0.9	16.6 ± 1.2	0.8
Preoperative To Postoperative Increase	1.7 ± 2.5	1.4 ± 2.8	0.601	1.8 ± 2.7	1.4 ± 2.4	0.569
Preoperative To Latest of Follow-Up Increase	2.8 ± 1.8	2.3 ± 1.5	0.453	3.3 ± 1.6	1.7 ± 1.4	0.004*
**Disc Height (mm)**						
Preoperative Value	6.5 ± 2.3	7.7 ± 2.4	0.047*	6.1 ± 2.5	7.7 ± 2.0	0.003*
Postoperative Value	8.6 ± 1.5	9.3 ± 1.2	0.053	8.7 ± 1.3	8.9 ± 1.6	0.596
Follow-Up Value	8.7 ± 1.7	9.0 ± 1.2	0.561	8.6 ± 1.4	9.0 ± 1.7	0.312
Preoperative To Postoperative Increase	2.1 ± 1.8	1.6 ± 1.6	0.278	2.6 ± 1.9	1.3 ± 1.4	0.001*
Preoperative To Latest of Follow-Up Increase	2.2 ± 2.0	1.2 ± 1.5	0.043*	2.6 ± 2.0	1.3 ± 1.6	0.023*
Latest Follow-Up To Postoperative Decrease (Subsidence)	0.13 ± 1.1	−0.35 ± 0.38	0.047*	−0.06 ± 0.9	0.02 ± 0.99	0.713
**Segmental Lordosis (°)**						
Preoperative Value	5.3 ± 3.1	6.2 ± 2.9	0.248	4.2 ± 3.0	6.9 ± 2.5	0.0001*
Postoperative Value	11.6 ± 4.8	10.4 ± 5.2	0.32	11.9 ± 4.7	10.6 ± 5.2	0.273
Follow-Up Value	7.1 ± 2.0	6.6 ± 1.4	0.266	6.8 ± 1.4	7.1 ± 2.2	0.514
Preoperative To Postoperative Increase	6.3 ± 5.6	4.1 ± 5.2	0.128	7.7 ± 5.2	3.7 ± 5.3	0.002*
Preoperative To Latest of Follow-Up Increase	1.8 ± 2.5	0.4 ± 2.3	0.025*	2.6 ± 2.7	0.2 ± 1.6	0.0001*
**CSA of Thecal Sac (mm**^**2**^**)**						
Preoperative Value	134.3 ± 23.5	142.5 ± 20.4	0.162	138.1 ± 24.3	135.8 ± 21.5	0.67
Postoperative Value	179.0 ± 14.6	176.3 ± 17.8	0.505	179.2 ± 16.0	177.2 ± 15.3	0.582
Follow-Up Value^a^	174.4 ± 10.8	172.3 ± 5.8	0.443	172.3 ± 8.9	175.1 ± 9.1	0.358
Preoperative To Postoperative Increase	44.7 ± 29.5	33.8 ± 26.3	0.142	41.1 ± 32.9	41.4 ± 24.9	0.969
Preoperative To Latest of Follow-Up Increase	38.2 ± 29.6	31.8 ± 24.3	0.494	35.2 ± 25.8	36.0 ± 30.1	0.932
**VAS Back Pain**						
Preoperative To the Last Follow-Up Improvement	2.33 ± 0.98	2.4 ± 0.6	0.738	2.2 ± 0.9	2.5 ± 0.8	0.214
**VAS Leg Pain**						
Preoperative To Last Follow-Up Improvement	2.6 ± 1.1	2.6 ± 0.9	0.900	2.5 ± 0.7	2.6 ± 1.0	0.622
**ODI**						
Preoperative To Last Follow-Up Improvement	20.7% ± 10.6%	20.4% ± 8.0%	0.912	19.0% ± 7.2%	19.2% ± 9.8%	0.929

*ODI* indicates Oswestry Disability Index, *VAS* Visual analog scores

* *P*<0.05 are considered statistically significant. ^a^ Corresponding radiographic parameters obtained from 26 cases with 36 operated levels with CT and MR images at the final follow-up

**Table 5:** Relationship of fusion patterns with the position and height of LLIF cages

	Circumferential fusion	Anterior fusion only	*P* value
Cage position			
Anterior	16	8	0.471
Posterior	6	6	
Cage height			
11-mm	15	8	0.723
13-mm	7	6	

Corresponding radiographic parameters obtained from 26 cases with 36 operated levels with CT and MR images at the final follow-up

Cages with a lower height were chosen for implantation in the operated intervertebral levels that showed lower height on preoperative radiographs. Preoperative, postoperative and final follow-up LL values in the 11-mm cage group were all lower than in the 13-mm cage group; however, the postoperative and final follow-up LL change in the 11-mm cage group was larger than in the 13-mm cage group (Table 4). Although preoperative FH, DH, and SA in the 11-mm cage group were significantly lower than in the 13-mm cage group, the two groups had similar FH, DH, and SA postoperatively and at the final follow-up. The mean FH at the final follow-up was larger by 3.3 mm in the 11-mm cage group, and 1.7- mm in the 13-mm cage group compared to the preoperative FH. The mean postoperative and final follow-up increases in DH and SA in 11-mm cage group were larger than in the 13-mm cage group (Table 4). The two cage height groups showed similar fusion patterns (Table 5).

### Clinical outcome measures

Clinical evaluation at the last follow-up revealed a mean ODI and back and leg VAS improvement of 19.0% ± 0.9%, 2.3 ± 0.9 points(48%), and 2.6 ± 1.0 points (58%), respectively. The clinical outcomes were similar between the respective subgroups in our study (anterior vs. posterior cage position; 11 vs. 13 mm cage height; *p* ≥ 0.05 for all; Table 4).

The surgeries were generally well tolerated, with no intraoperative complications, no cases of femoral nerve paralysis, or bowel injury. Non-neurological inpatient complications included one case (2.1%) of myocardial ischemia. Six patients (12.8%) experienced postoperative thigh numbness, which resolved over the first three months after surgery in all cases.

## Discussion

This study demonstrated excellent clinical results in patients treated by LLIF that efficiently restored their radiographic parameters. The anterior cage position was associated with the most significant SA and DH correction, while the cages in posterior position seemed to increase the incidence of the cage subsidence. Selection of the proper cage height based on preoperative radiographic parameters improved the probability of lumbar alignment restoration.

Consistent with previous studies [4, 15], we found no impact for cage position on LL, FH, and CSA. Alimi et al. [15] showed that the cage positioning did not affect the radiographic outcomes, including LL and FH. Kepler et al. [4] evaluated change in foraminal area after XLIF and reported that the foraminal area and disc height were not influenced by the cage position. However, studies reported by Hiyama et al. [21] and Kepler et al. [22] showed that the more anterior the cage position was, the greater the LL that was obtained, suggesting that the cage position of the cage might affect the radiographic parameters. Similarly, we found the SA and DH were influenced by the cage position in this study. SA was restored in the anterior cage position as it was significantly larger than in the posterior position. These findings were corroborated by Shiga and colleagues [23], who concluded that SA increased with how far anteriorly the cage was positioned following oblique lateral interbody fusion (OLIF). Although DH values in the posterior and anterior positions were similar immediately postoperatively and at the final follow-up, the magnitude of improvement in the anterior cage position group was significantly larger than in the posterior cage position group, indicating that anteriorly positioned cages were better tolerated in intervertebral disc space restoration. These findings remind us of the LLIF cages should be implanted in the anterior intervertebral position during LLIF to obtain a more stable structure.

In this study, the appropriate height of the cages was implanted based on the preoperative intervertebral disc space. The preoperative DH, FH, SA, and LL values in the 11-mm cage height group were smaller than those in the 13-mm cage height group; however, the group had similar DH, FH, and SA but not LL immediately postoperatively and at 12 months later, demonstrating the strong lumbar alignment restoration of the cage and the importance of its proper cage height in the LLIF procedure. Previous study demonstrated that optimal cage height in LLIF was important because the postoperative increase in disc height and the disc height loss at the final follow-up were closely associated [24]. Barone et al. [9] demonstrated that disc angle and lumbar lordosis restorations were effective because lordotic cages were utilized in the LLIF, with the improvement magnitude significantly higher in the lower cage height group for DH, FH, SA, and LL immediately postoperatively and at final follow-up. Landham et al. [25] concluded that a moderate cage height should be implanted since higher cages might overstuff the disc space and decrease the extent of the lumbar monosegemental lordosis in posterior lumbar interbody fusion (PLIF) surgery. They suggested that the contradictory radiographic outcomes could be because the anterior longitudinal ligament might act as a tether and restricting the magnitude of radiographic outcomes intraoperatively when a tall cage was used [25]. The immediate postoperative radiographic cage height outcomes were similar in all cases. Furthermore, the lumbar lordosis at the postoperative and final follow-up was significantly higher in the 13-mm cage group than in the 11-mm cage group, suggesting a cage height effect and its superior capabilities in correction of lumbar spine alignment.

Cage subsidence was observed in this study. Tomeh et al. [26] reported that higher implanted cages were significantly associated with an increased risk of cage subsidence following the XLIF procedure. However, in this study, we found no difference between the two intervertebral cage height groups. Conversely, the cage position correlated with the cage subsidence, showing more subsidence in posteriorly than anteriorly positioned cages. Contrary to our findings, Alimi et al. [15] found no difference in the amount of subsidence between the anteriorly and posteriorly positioned cages. The difference in subsidence could be attributable to that the posterior vertebral endplate is relatively weak [27] and posterior cages spanned less of the endplate ring apophysis than the anterior cages due to the special shape of the vertebrates [14, 28]. Therefore, we suggest placing the cage at least in the anterior half of the disc space to reduce the risk of cage subsidence following LLIF.

The LLIF cage position and height had no effect on the fusion rate and patterns in the current study. We noted a fusion rate of 84.3% and a circumferential fusion rate of 61.1% at the 1-year follow-up, similar to previous studies [20, 29]. The lack of difference could be because the likelihood of bone growth and fusion success was enhanced by implanting cages with a wide contact area, interbody grafting immediately reduced motion by an average of 70% [30] regardless of the cage position, and the LLIF technique ensured compressive loading and proper cage height by preserving the longitudinal ligament [29].

This study had several limitations. The sample size was small and its sex ratio is imbalanced, therefore, more participants and a balanced sex ratio are warranted in the future. As a retrospective study, it had inherent limitations, such as missing and/or incomplete data. Last follow-up MR and CT scans were available only for some participants, limiting the availability of some radiographic parameters such as FH and CSA, possibly decreasing the strength of the conclusions. We did not assess the bone mineral density. Age-related osteoporosis may have affected the occurrence of cage subsidence. Furthermore, we used only two cage height types; further studies will need to clarify the effects of other cage heights on the radiographic parameters. And finally, it was performed at a single center, and a comparative analysis of cage widths and other potentially relevant factors was not performed.

## Conclusions

LLIF is a feasible and efficient approach in the treatment of lumbar degenerative diseases. Cage height and positioning do not have a determining role in clinical outcomes. Anteriorly positioned cages showed better in DH and SA restoration and lower incidence of cage subsidence; therefore, during the LLIF procedure, the position of the cage should be implanted anteriorly. The proper height of the cage should be selected based on preoperative radiography for positive radiographic outcomes following LLIF.

## Data Availability

The datasets generated and/or analyzed during the current study are not publicly available due to limitations of ethical approval involving the patient data and anonymity but are available from the corresponding author on reasonable request.

## Abbreviations

LLIF: Lateral lumbar interbody fusion
LL: Lumbar lordosis
FH: Foraminal height
DH: Disc height
SA: Segmental angle
CSA: Cross-sectional area
ODI: Oswestry Disability Index
Vas: Visual analog scale
TLIF: Transforaminal lumbar interbody fusion
PLIF: Posterior lumbar interbody fusion
XLIF: Extreme lumbar interbody fusion
PEEK: Polyether-ether-ketone
MR: Magnetic resonance
SD: Standard deviation
OLIF: Oblique lateral interbody fusion
